# Use of bispectral index EEG monitoring for a fast and reliable detection of epileptic activity in postcardiac arrest patients

**DOI:** 10.1186/cc14514

**Published:** 2015-03-16

**Authors:** J Haesen, L Desteghe, I Meex, C Genbrugge, J Demeestere, J Dens, L Ernon, C De Deyne

**Affiliations:** 1 Universiteit Hasselt, Belgium; 2 Ziekenhuis Oost-Limburg, Genk, Belgium

## Introduction

Assessment of prognosis in postcardiac arrest (post-CA) patients became very challenging since the Introduction of therapeutic hypothermia (TH). Continuous EEG monitoring has been proposed to improve prognostication; however, its use is limited due to difficulties in ready interpretation. Thus emerges the need for a simple EEG montage. The bispectral index (BIS) monitor is a simplified EEG system, mainly calculating an index ranging from 0 (isoelectric EEG) to 100 (full consciousness) to provide information on hypnotic depth of anaesthesia. The aim of the study was to validate the accuracy of simplified EEG monitoring in a CA setting.

## Methods

BIS monitoring (BIS VISTATM) was applied to collect frontotemporal data in TH-treated CA patients. A standard 19-channel EEG was performed after return to normothermia. Afterwards, small EEG frames coincident with the time of full EEG registration were extracted from the BIS monitor. We asked two neurophysiologists to indicate the presence of status epilepticus (SE), cerebral inactivity (CI), burst suppression (BS), periodic epileptiformic discharges (PEDs) or a diffuse slowing pattern (DS). In addition, these samples were analysed by two inexperienced physicians, who were asked to indicate the presence of SE.

## Results

Thirty-four simplified EEG samples were analysed. According to standard EEG, 11 patients showed a DS pattern, three had CI, six showed BS, four showed PEDs and 10 had an SE. Neurophysiologists interpreted all samples with a high accuracy. Mean sensitivity was 82.12% and mean specificity was 91.88%. Only one SE was missed by one neurophysiologist. Unfortunately, only one PED was confirmed by both neurophysiologists. Interobserver reliability was high (κ = 0.843). High correlations were found for the comparison of full and simplified EEG for both neurophysiologists (*r *= 0.809). Further, the two inexperienced physicians identified SE with a sensitivity of 85% and specificity of 98%.

## Conclusion

Simplified EEG monitoring, using BIS, resulted in high accuracy of a simple classification system in post-CA patients. Not only neurophysiologists, but also treating physicians were capable to identify SE, which may play an important role in the early detection of SE. We suggest using BIS as a screening tool in post-CA patients to save valuable time in the detection of SE, without replacing the need for full EEG monitoring for confirmation.

